# The Journal Citation Indicator has arrived for Emerging Sources Citation Index journals, including the *Journal of Educational Evaluation for Health Professions*, in June 2021

**DOI:** 10.3352/jeehp.2021.18.20

**Published:** 2021-08-02

**Authors:** Sun Huh

**Affiliations:** Department of Parasitology and Institute of Medical Education, College of Medicine, Hallym University, Chuncheon, Korea; The Catholic University of Korea, Korea

## The appearance of the Journal Citation Indicator

On June 30, 2021, I was notified that the 2020 Journal Citation Ranking (JCR) had been published. The most striking feature was a new citation index, the Journal Citation Indicator (JCI). Szomszor [1] announced on Clarivate’s blog, “The JCI provides a single journal-level metric that can be easily interpreted and compared across disciplines. The value of the JCI is the mean Category Normalized Citation Impact (CNCI) for all articles and reviews published in a journal in the preceding 3 years” [1]. The main difference between the JCI and the Journal Impact Factor (JIF) is that the denominator of the JCI is the number of reviews and articles for only the most recent 3 years, while the numerator is the citations of those reviews and articles for 4 years, including the current year. A JCI value of 1.0 corresponds to the mean value of each category of journals in the Web of Science Core Collection. A JCI value of 2 indicates that the citation impact of a journal in the corresponding category is 2 times the mean value., whereas a JCI value of 0.5 means that a journal’s impact is half of the mean value in the corresponding category.

## *Journal of Educational Evaluation for Health Professions’* performance

**Fig. 1.**
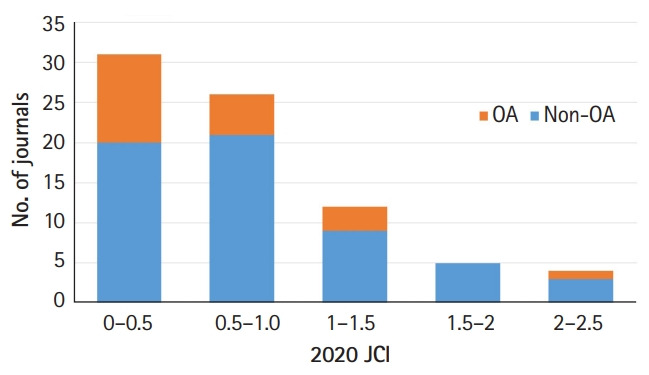
Number of journals according to the 2020 Journal Citation Indicator (JCI) in the category of scientific education in the 2020 Journal Citation Report. OA, open-access journals; non-OA, non-open-access journals.

*Journal of Educational Evaluation for Health Professions* (JEEHP) received a JCI value of 0.51. Fig. 1 shows the number of journals in the scientific education category of the 2020 JCR according to their 2020 JCI. This list includes 43 Science Citation Index Expanded (SCIE) and 35 Emerging Sources Citation Index (ESCI) journals. One SCIE journal was excluded since it received no JCI value. Out of these 78 journals, 20 journals were published as open access. The minimum value of the 2nd quartile of the 2020 JIF was 2.333. The highest value of ESCI journals’ JCI was 0.82. Therefore, JEEHP’s ranking was 9th out of 35 ESCI journals (74.3%) and 47th out of 78 SCIE and ESCI journals (39.8%). Potentially, this result is relatively good for an ESCI journal, but indicates that JEEHP still has room for improvement in the context of SCIE journals

The manually calculated 2020 JIF of JEEHP from the Web of Science Core Collection was 1.254 (79/63). The number of citable articles published from 2018 to 2019 was 63, and the number of citations of all articles published from 2018 to 2019 in the 2020 Web of Science Core Collection was 79. This value corresponds to 34th place out of 44 (24.5%) SCIE journals in the category. The Pearson correlation coefficient between the 2021 JCR and JCI of the 43 SCIE journals in the category of scientific education was 0.9005 (P<0.0001; 95% confidence interval, 0.8227–0.9452).

## How to cope with the struggle for citations

Many scientific groups do not recommend evaluating articles in terms of journal-level citation indicators, including the JIF. The quality of an article itself reflects the essential value of the research. However, journal brands are easily recognized based on the JIF or other citation indicators, including the Eigenfactor score, Article Influence Score, CiteScore, Source Normalized Impact per Paper, SCImago Journal Rank, and Hirsch index [2]. At many universities in Korea, the JIF has even been used to measure faculty members’ competency as part of faculty appointments and promotion evaluations. This is also the case at my university, Hallym University in Korea. There is no reason to expect that these evaluation policies will change in the near future due to the lack of other objective measurement tools.

JEEHP also has reasons to consider the citation frequency. Some submitters have inquired whether JEEHP is an SCIE journal. Although it has been indexed in international databases, including PubMed, PubMed Central, MEDLINE, Scopus, EMBASE, Web of Science Core Collection, and ESCI, the impact of being an SCIE journal is tremendous. Therefore, the editorial team has regularly checked JEEHP’s citation frequency through Crossref metadata. The 4 most frequently cited articles published from 2019 to 2020 and their citation frequencies as recorded by Crossref are as follows: an editorial on personal protection from SARS-CoV-2 (severe acute respiratory syndrome coronavirus 2) infection, 78 citations [3]; a review article on the artificial intelligence in medical education, 17 citations [4]; a research article on the factors influencing the career preferences of medical students and interns, 7 citations [5]; and a review article on management guidelines for extravasation, 7 citations [6].

It is difficult to estimate the likelihood that specific articles will receive many citations during the review and publication process. It was astonishing to view the citations of the above-mentioned editorial, which is a guideline for the self-protection of health personnel. The 2 review articles listed above were commissioned due to their importance for education in the future and the lack of freely accessible detailed guidelines. The research article was unsolicited. There is no other way to increase citations except by reviewing manuscripts meticulously for their scientific integrity and usefulness.

In addition to publishing high-quality articles, JEEHP has maintained publishing policies that adhere to the guideline on transparency and best practice in scholarly publishing since 2018. However, some SCIE journals in Korea and other countries worldwide have still not complied with this best practice [7,8]. This best practice may not be mandatory for scholarly journals; however, JEEHP adopted it to be regarded as a scholarly journal that goes beyond the minimum requirements. An open-access policy is mandatory and inevitable as a way for local institutional journals to increase their visibility and citations. This point leads to the following question: what is the proportion of gold open-access journals in JCR? Out of 12,449 journals in the 2019 JCR, 1,640 journals were gold open-access (13.1%) [9]. This proportion increased slightly in the 2020 JCR, as shown in Table 1. If only SCIE and SSCI journals are considered, the proportion of open-access journals is 14.5% (1,788/12,312).

As shown in Table 1, ESCI has the highest proportion of open-access journal titles compared to other citation indexes. However, there may be more open-access journals that are not listed in DOAJ (Directory of Open Access Journals; https://doaj.org/). This new phenomenon of open-access journal publication may be a continuing trend in the scholarly journal market. Commercial publishing companies that only publish open-access journals have increased the number of journal titles. Traditional commercial publishing companies have also begun to publish open-access journals, upon recognizing that it is possible to operate companies on a commercially sustainable basis by receiving article processing charges from authors without library subscription fees. Out of 20 gold open-access journals in the 2020 JCR scientific education category, JEEHP is one of 8 diamond open-access journals, which means that the journal does not require an article processing charge. This policy aims to remove economic barriers to submission.

JEEHP has also adopted a data-sharing policy. In 2019, 13 journals in Korea had adopted a data-sharing policy, and 10 of these journals framed this policy as only a recommendation. Two journals adopted an optional data deposit policy. Only 1 journal (JEEHP) adopted mandatory deposition and peer review of data [10].

## Relevance of the JCI for ESCI journal editors and publishers

Although it is possible to calculate the impact factor of ESCI journals manually, the JCI serves as an official indicator of ESCI journals. Therefore, it provides an excellent opportunity to compare ESCI journals with other journals in SCIE and ESCI regardless of category, since its value is based on the mean CNCI for all articles and reviews. With an eye towards indicators such as the JCI, editors will increasingly focus on recruiting high-quality reviews and research articles.

## Genuine wish

Of note, 150 out of the 163 total manuscript submissions (92.0%) to JEEHP from January to July 11, 2021 were from authors outside Korea. The editorial team will do its best to review manuscripts meticulously and rapidly and recruit informative articles. I appreciate the authors, reviewers, and readers for their devotion and advice with the goal of making JEEHP competitive. Their voluntary work and dedication made it possible for JEEHP to receive its first JCI. Since we are still in the COVID-19 (coronavirus disease 2019) pandemic era, I would like to extend my genuine wish for the journal’s readers, reviewers, and authors to be safe and healthy.

## Figures and Tables

**Table 1.** The proportion of gold open-access journals in 2020 Journal Citation Reports (cited 2021 July 11)

	No. of journals	No. of DOAJ journals (%)
SCIE	9,500	1,611 (17.0)
SSCI	3,510	268 (7.6)
A&HCI	1,784	109 (6.1)
ESCI	7,275	2,797 (38.4)
Total^a)^	20,932	4,672 (22.3)

DOAJ, Directory of Open Access Journals (https://doaj.org/); SCIE, Science Citation Index Expanded; SSCI, Social Science Citation Index; A&HCI, Arts and Humanity Citation Index; ESCI, Emerging Sources Citation Index.

a) The total number is different from the sum of journals of each citation index area because some journals included 2 areas among SCIE, SSCI, and A&HCI.
